# The Tuberculin Test

**Published:** 1900-02-17

**Authors:** 


### THE TUBERCULIN TEST.

Some experiments which have lately been made at the instance of the Technical Instruction Committee of the Cheshire County Council, with the object of determining certain points in regard to the use of tuberculin as a test for tuberculosis in cattle, have led to a result which, although by no means surprising to a bacteriologist, is of considerable importance in reference to the dependence which is in some quarters placed upon this method of diagnosis. Nine cows were isolated and submitted to repeated injections of tuberculin at certain intervals, and were afterwards slaughtered and examined, and from the results obtained the conclusion was arrived at that " continuous injections of tuberculin at short periods of time will cause reaction to cease in an animal that has previously been shown to be tuberculous." It is easy to see then that if tuberculin were to come into general use commercially as a test in the cattle market, or if the custom were to extend among milk dealers of demanding a guarantee that the milk supplied is derived from tuberculin-tested cows, an easy means of fraud would be in the hands of anyone who chose, by repeated injections, to render his diseased cows immune to the test.

